# Specific CD4+ T Cell Responses to Ancestral SARS-CoV-2 in Children Increase With Age and Show Cross-Reactivity to Beta Variant

**DOI:** 10.3389/fimmu.2022.867577

**Published:** 2022-07-15

**Authors:** Kevin Paul, Freya Sibbertsen, Daniela Weiskopf, Marc Lütgehetmann, Madalena Barroso, Marta K. Danecka, Laura Glau, Laura Hecher, Katharina Hermann, Aloisa Kohl, Jun Oh, Julian Schulze zur Wiesch, Alessandro Sette, Eva Tolosa, Eik Vettorazzi, Mathias Woidy, Antonia Zapf, Dimitra E. Zazara, Thomas S. Mir, Ania C. Muntau, Søren W. Gersting, Gabor A. Dunay

**Affiliations:** ^1^ University Children’s Research - UCR@Kinder-UKE, University Medical Center Hamburg-Eppendorf, Hamburg, Germany; ^2^ Department of Pediatrics - Kinder-UKE, University Medical Center Hamburg-Eppendorf, Hamburg, Germany; ^3^ Center for Infectious Disease and Vaccine Research, La Jolla Institute for Immunology, La Jolla, CA, United States; ^4^ Institute of Medical Microbiology, Virology and Hygiene, University Medical Center Hamburg-Eppendorf, Hamburg, Germany; ^5^ German Center for Infection Research, partner site Hamburg-Lübeck-Borstel-Riems, Hamburg, Germany; ^6^ Institute of Immunology, University Medical Center Hamburg-Eppendorf, Hamburg, Germany; ^7^ First Department of Medicine, Division of Infectious Diseases, University Medical Center Hamburg-Eppendorf, Hamburg, Germany; ^8^ Department of Medicine, Division of Infectious Diseases and Global Public Health, University of California, San Diego (UCSD), La Jolla, CA, United States; ^9^ Institute of Medical Biometry and Epidemiology, University Medical Center Hamburg-Eppendorf, Hamburg, Germany; ^10^ Division for Experimental Feto-Maternal Medicine, Department of Obstetrics and Prenatal Medicine, University Medical Center Hamburg-Eppendorf, Hamburg, Germany; ^11^ Department of Pediatric Cardiology, University Medical Center Hamburg-Eppendorf, Hamburg, Germany

**Keywords:** peptide, variant of concern (VOC), ancestral, activation induced marker (AIM), human coronavirus (HCoV), pediatric, COVID-19

## Abstract

SARS-CoV-2 is still a major burden for global health despite effective vaccines. With the reduction of social distancing measures, infection rates are increasing in children, while data on the pediatric immune response to SARS-CoV-2 infection is still lacking. Although the typical disease course in children has been mild, emerging variants may present new challenges in this age group. Peripheral blood mononuclear cells (PBMC) from 51 convalescent children, 24 seronegative siblings from early 2020, and 51 unexposed controls were stimulated with SARS-CoV-2-derived peptide MegaPools from the ancestral and beta variants. Flow cytometric determination of activation-induced markers and secreted cytokines were used to quantify the CD4+ T cell response. The average time after infection was over 80 days. CD4+ T cell responses were detected in 61% of convalescent children and were markedly reduced in preschool children. Cross-reactive T cells for the SARS-CoV-2 beta variant were identified in 45% of cases after infection with an ancestral SARS-CoV-2 variant. The CD4+ T cell response was accompanied most predominantly by IFN-γ and Granzyme B secretion. An antiviral CD4+ T cell response was present in children after ancestral SARS-CoV-2 infection, which was reduced in the youngest age group. We detected significant cross-reactivity of CD4+ T cell responses to the more recently evolved immune-escaping beta variant. Our findings have epidemiologic relevance for children regarding novel viral variants of concern and vaccination efforts.

## Introduction

SARS-CoV-2 appeared in 2019 (1) causing a pandemic which, despite effective vaccination, is still a major threat to global health (2). Children in particular are now facing increasing infection rates due to a reduction of social distancing measures while vaccination of younger age groups has just begun or is not yet available. So far, the disease course in the younger population appears to be mild (3, 4), however emerging variants may present new challenges. With the current emerging omicron, variant hospitalization rates of young children in particular appear to be on the rise (5). Information on the pediatric immune response after infection or vaccination is of great importance for planning protective strategies in the future. However, data on T cell-mediated immunity in children is still lacking. The identification of antigen-specific T cells via stimulation of patient PBMC with viral peptide pools followed by detection of reactive T cells through activation-induced markers allows the identification and simultaneous phenotyping of these cells using limited available patient material (6) and has been broadly used to identify SARS-CoV-2 reactive T cells in adults (7–16), and children (17, 18). The COVID-19 Child Health Investigation of Latent Disease (C19.CHILD) Hamburg Study recruited children from all age groups after the spring 2020 wave of SARS-CoV-2 infections in Hamburg, Germany. Here, PBMC from over fifty SARS-CoV-2 convalescent children, their exposed siblings and unexposed age-matched controls from the C19.CHILD cohort were stimulated with peptide MegaPools (MP) spanning the entire SARS-CoV-2 Spike Glycoprotein of the Wuhan-Hu-1 strain and beta variant as well as predicted peptides representing the remaining entire SARS-CoV-2 Wuhan-Hu-1 strain proteome (19) to detect and characterize virus-specific CD4+ T cell responses.

## Materials and Methods

### Study Cohort and Ethics

SARS-CoV-2 convalescent children, exposed seronegative siblings as well as unexposed controls were identified from the COVID-19 Child Health Investigation of Latent Disease (C19.CHILD) Hamburg Study cohort, registered at clinicaltrials.gov (NCT04534608). Briefly, 6113 children (<18 years) who presented voluntarily or were recruited while receiving care in one of the five pediatric hospitals of Hamburg, Germany, were invited for a screening for an acute or recent SARS-CoV-2 infection *via* PCR and serum antibody testing.

Patients who tested positive in the PCR and/or the antibody screening were invited with all household members for a follow-up appointment, where detailed history was obtained and PCR and serologic SARS-CoV-2 testing were repeated. PBMC samples were obtained from all family members under 18 years. Pediatric unexposed and healthy volunteers, with no known SARS-CoV-2 contact, were welcomed to enrol through the C19.CHILD Study Clinic.

Recruiting was conducted from May 11^th^ until June 30^th^ 2020 after the first infection wave, during and after the first lockdown in Germany. Parents or legal guardians provided written informed consent in all cases. From children over 7 years, consent in writing was obtained whenever possible but also consent in spoken word was accepted. The study was approved by the local ethical committee of Hamburg (reference number: PV7336).

### Serum Antibody Measurements

For screening purposes, serum samples were tested for SARS-CoV-2 specific antibodies directed against the viral nucleocapsid (IgA/IgM/IgG) using Elecsys^®^ Anti-SARS-CoV-2 Ig assay (Roche) on the cobas e411 system (Roche). Additionally, serum samples were tested for SARS-CoV-2 specific antibodies against the S1 and S2 subunits of the viral Spike protein using LIAISON^®^ SARS-CoV-2 IgG serology test (DiaSorin).

To evaluate serostatus for “common cold” coronaviruses (HCoV) and to further confirm SARS-CoV-2 serostatus, serum antibodies (IgG) against the viral nucleocapsid of HCoV strains 229E, NL63, OC43, HKU1 and SARS-CoV-2 anti - S1 subunit, anti - receptor binding domain as well as anti - nucleocapsid were measured by using the recomLine SARS-CoV-2 IgG^®^ assay (Mikrogen).

Antibody screening was performed IVD conform according to the manufacturer’s instructions.

Since patient recruiting was performed in a low prevalence setting, (<5%), we used an orthogonal testing algorithm. Therefore, samples were considered as SARS-CoV-2 positive if all three tests were positive, negative in three tests was considered as SARS-CoV-2 negative. Participants with discordant results were excluded from later analysis. Unexposed controls were required to report no known SARS-CoV-2 exposure and to be negative in the SARS-CoV-2 PCR test and in the three antibody tests.

### Sample Acquisition and Processing

Pediatric blood samples (2-10 ml) were collected in EDTA and processed within 24 hours. PBMC were isolated by gradient centrifugation using SepMate tubes^®^ and Lymphoprep^®^ (StemCell) according to the manufacturer’s instructions. PBMC were cryopreserved in freezing medium containing 50% FBS (Capricorn), 30% RPMI (Gibco) and 20% DMSO (AppliChem) and stored in liquid nitrogen until further analysis.

For analysis, frozen aliquots of PBMC were incubated for one minute in a 37°C water bath, and subsequently thawed in prewarmed RPMI by gentle pipetting.

### PBMC Peptide Stimulation

Peptide stimulation was conducted using previously described (12, 19) peptide MegaPools optimized for stimulation of CD4+ T cells spanning the entire SARS-CoV-2 spike glycoprotein (Spike-OS_MP) as well as predicted peptides representing the remaining entire SARS-CoV-2 proteome (R_MP) of the first described original strain (Wuhan-Hu-1). Additionally, a peptide pool of overlapping 15-mer by 10 amino acids covering the entire SARS-CoV-2 beta variant spike glycoprotein (Spike-BV_MP) was used. The compositon of the Spike-BV_MP was similar to the original strain peptide pool, only peptides containing beta variant specific mutations were exchanged. Beta variant specific peptides according to respective mutations as well as peptide pool synthesis were as described (15).

Thawed PBMC were incubated in 5ml RPMI + human serum (Pan Biotech) 5% + Benzonase (Sigma-Aldrich) 50 U/ml for 1 hour (37°C 5% CO_2_), followed by a washing step with 15ml RPMI + human serum (HS) 5%. Afterwards all available cells were equally divided to be stimulated for 24 hours (37°C 5% CO_2_) in 200µl RPMI + HS 5% in 96-well U-bottom plates with mentioned peptide MegaPools (1µg/ml/peptide), PHA-L (Invitrogen) (1µg/ml) as positive control and an equimolar amount of DMSO to serve as negative control. After 24h of stimulation, cell culture supernatant was carefully removed and stored at -20°C for later multiplex cytokine analysis. Incubation was stopped by washing cells in PBS. Expression of activation-induced markers (CD69 and OX40) in response to specific peptide stimulation, as well as their memory phenotype were measured by flow cytometry (flow cytometry antibodies are listed in **Table S1**).

### Flow Cytometry

After thawing, samples for ex-vivo immune phenotyping were washed twice with PBS, equally split into two aliquots, and analyzed with two optimized panels for investigating T cell, B cell and innate immune cell phenotypes, established previously and slightly adapted (20).

The staining procedure for ex-vivo immune phenotyping as well as for PBMC after peptide stimulation was as follows: Cells were stained with Near-IR Dead Cell Stain Kit (Invitrogen) incubated for 15 minutes in the dark at room temperature (RT) and subsequently stained with an antibody cocktail (**Table S1, Figure S1**) for 20 minutes at RT. Following washing with PBS, cells were fixed in 1% PFA (Morphisto) for one hour at 4°C, which was removed by PBS wash. Cells were kept at 4°C until acquisition.

Comparability of fluorescence intensities was regularly tested with Rainbow Calibration Particles (BD Sphero). For compensation, Anti-Mouse or Anti-Rat Ig, κ/Negative Control Compensation Particles Set (BD Biosciences) was used for antibodies, and ArC™ Amine Reactive Compensation Beads (Invitrogen) were used for the Dead Cell Stain Kit. Representative gating strategies are shown in **Figure S1**.

Antibody concentrations to achieve optimal separation of targeted populations were evaluated by titration in preliminary experiments using anonymous buffy coats, obtained at the Department of Transfusion Medicine at the University Medical Center Hamburg-Eppendorf from adult blood donors, who provided their written informed consent.

All flow cytometry measurements were performed with a BD FACSymphony A3 flow cytometer in the Cytometry and Cell Sorting Core Unit at University Medical Center Hamburg-Eppendorf.

### Multiplex Detection of Cytokines

Detection of cytokines in the cell culture supernatant of stimulated cells was performed using LEGENDplex™ Human CD8/NK Panel (13-plex, BioLegend) suitable for detection of IL-2, IL-4, IL-10, IL-6, IL-17A, TNF-α, sFas, sFasL, IFN-γ, Granzyme A, Granzyme B, Perforin, Granulysin, according to the manufacturer’s instructions. Briefly, freshly prepared provided cytokine standard or thawed cell culture supernatant was mixed with cytokine-specific beads, incubated for 2 hours and washed. After sequential incubation of bead-bound cytokines with biotin-labeled cytokine detection antibodies and streptavidin PE antibodies, non-binding antibodies were washed off and PE-labeled bead-bound cytokines were subsequently analyzed by flow cytometry. Quantification of cytokines was carried out using the standard. The data was analyzed using the online LEGENDplex™ Data Analysis Software of the manufacturer. The assay was performed in duplicates and mean values of each sample were used for further analysis.

### Data Analysis and Statistics

Data analysis, graphs and statistics were prepared with FlowJo version 10, and R 4.0.5 (packages: tidyverse, rstatix, splines, emmeans, kableExtra, magrittr, heatmaply). Paired sample analyses were performed by paired t-test. The stimulation index and fold increases showed highly skewed distributions and were therefore log-transformed for further analysis. An unpaired t-test was used for comparing two groups. Categorical variables were compared using Fisher’s exact test. Comparisons between three groups were done with one-way ANOVA and *post hoc* pairwise t-tests, if the ANOVA-F test was significant, thus following the closed test principle, no adjustment for multiple testing was necessary for pairwise comparisons. The association between age and the stimulation index was explored using a non-parametric spline regression with age and serology group and sex as independent variables. An interaction between spline age and serology group was initially included in the model, but it was removed from the final model, if it did not significantly increase the model fit. A p-value < 0.05 was considered as statistically significant in all analyses. As this study had an exploratory nature, we refrained from adjusting for multiple testing.

For analysis of flow cytometry experiments, samples with less than 5000 acquired alive CD3+ cells (using the DMSO control as reference for peptide stimulations) were excluded from further analysis.

Analysis of AIM+ cells was conducted as described previously (11–13), by calculating of a stimulation index by dividing the frequency of OX40+CD69+ cells within CD4+ cells after peptide stimulation with the frequency of the same cell subset in the negative control (DMSO). In case no OX40+CD69+ cells were detected with DMSO, the lowest detected frequency for DMSO in the given experimental group was used.

## Results

### Study Cohort Characteristics

The study cohort consisted of 126 participants: 51 seropositive children as determined by positivity in all of three separate serological tests covering the viral Spike and Nucleocapsid, 24 seronegative siblings living in a shared household with an infected individual and 51 age- and gender-matched unexposed controls. There were no significant differences in the age- and gender distribution of these groups (**Table 1**). For children whose families were able to give a detailed account of past infection (68 of 75 seropositive children and seronegative siblings), 55% of seropositives and 43% of seronegative siblings reported symptoms consistent with COVID-19. Most families with several symptomatic children could no longer recall the exact date of symptom onset (DSO) for each individual child. However, for 26 out of 35 symptomatic children, families were able to recall DSO of the first symptomatic child in the household which we applied as an approximation to all siblings. The mean time since DSO at the time of sampling was similar within the two groups: 84 days (range 51 – 115 days) for seropositives and 83.4 days (range 62 – 103 days) for seronegative siblings.

**Table 1:** Table showing basic characteristics of the cohort.

	Seropositives (n=51)	Seroneg. siblings (n=24)	Unexp. controls (n=51)	p	Test
**Age (years, mean ± range)**	10.63 (0 - 17)	8.79 (1 - 16)	10.78 (1 - 17)	0.170	ANOVA
**Sex (female)**	21 (41%)	12 (50%)	27 (53%)	0.523	Fisher’s exact
**HCoV serology (positive)**	25 (49%)	8 (33%)	18 (35%)	0.291	Fisher’s exact
** **	**Seropositives (n=47)**	**Seroneg. siblings (n=21)**			
**Symptoms (yes)**	26 (55%)	9 (43%)		0.433	Fisher’s exact
** **	**Seropositives (n=21)**	**Seroneg. siblings (n=5)**			
**Days PSO (mean ± range)**	84 (51 - 115)	83.4 (62 - 103)		0.907	t-test

Number of participants (n) per each analysis and subgroup is indicated as data was available. Groups according to SARS-CoV-2 serology: seropositive, seronegative (exposed) siblings, unexposed controls. The last two columns describe the statistical analyses per row, the p values were calculated with the statistical test as indicated in the last column. HCoV: human “common cold” coronaviruses 229E, NL63, OC43, HKU1. PSO: days post-symptom onset of the first symptomatic child in the family.

SARS-CoV-2 seropositive study participants were all convalescent of an ancestral variant, closely related to the original Wuhan-Hu-1 strain, since recruiting took place before the occurrence of the first SARS-CoV-2 variants of concern.

### Antigen-Specific CD4+ T Cells After Infection With an Ancestral SARS-CoV-2 Variant

**Figure 1:**
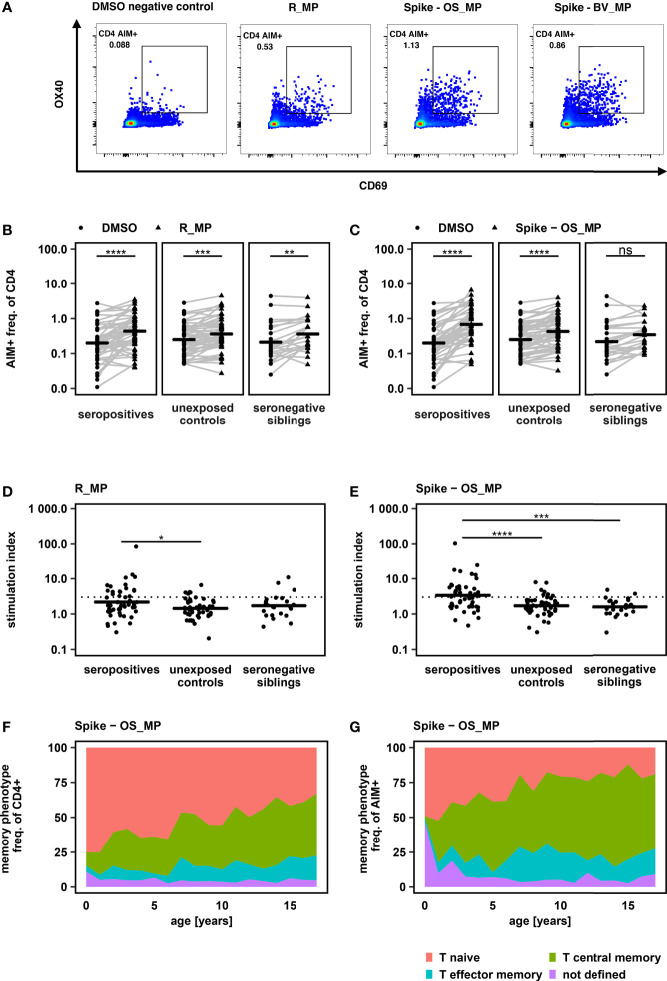
**(A)** Flow cytometry results of a representative SARS-CoV-2 seropositive study participant, gated on alive CD3+ CD4+ lymphocytes. PBMC were equally divided and stimulated over 24h with either DMSO (negative control), PHA-L (positive control) or SARS-CoV-2 derived peptide MegaPools covering the spike glycoprotein of the original strain Wuhan-Hu-1 (Spike-OS_MP), beta variant (Spike-BV_MP) or the remaining original strain’s proteome (R_MP). CD4 AIM+ T cells were identified by their CD69 and OX40 expression. CD4 AIM+ gate was set equal for all samples of all participants after comparability of fluorescence intensities was assured by rainbow bead calibration. **(B + C):** Frequency of AIM+ cells, determined by CD69 and OX40 expression, after stimulation with R_MP and Spike -OS_MP in comparison to AIM+ frequency after DMSO exposure. Study groups were determined by their SARS-CoV-2 serostatus and SARS-CoV-2 exposure. Horizontal lines represent mean values. One sided paired t – tests were used to determine P values. **(D + E)**: Comparison of T cell response towards peptide stimulation between study groups. T cell response was quantified by using a Stimulation Index (SI) which was calculated by dividing the freq. of AIM+ cells after peptide stimulation by the freq. of AIM+ cells of the DMSO negative control. A SI > 3 (dashed line) was defined as response. Mean values are shown as horizontal lines. Statistical comparisons were carried out by one way ANOVA and *post hoc* pairwise t – tests. **(F + G)**: Memory phenotype of total CD4+ and AIM+CD4+ T cells after peptide stimulation with Spike - OS_MP. Mean values of all study participants irrespective of SARS-CoV-2 serostatus are displayed *P < 0.05, **P < 0.01, ***P < 0.001, ****P < 0.0001, ns, not significant.

PBMC from all donors were stimulated with peptide MegaPools derived from the first described original SARS-CoV-2 strain (Wuhan-Hu-1) spanning the entire Spike glycoprotein (Spike-OS_MP) and predicted epitopes from the remaining proteome (R_MP). CD4+ T cell response was measured based on the expression of AIM markers (OX40 and CD69) and compared to a negative control consisting of the peptide mix solvent DMSO at the same concentration as in the peptide mix (**Figures 1A**, **S1A**). When compared to the carrier control (DMSO), stimulation with R_MP led to an increase in the expression of AIM markers in all groups. Increased expression of AIM markers after Spike-OS_MP stimulation was detectable in the seropositive and unexposed control, but not in the seronegative group (**Figures 1B, C**). This reflects a combination of antigen-specific CD4+ T cell activation together with an additional unspecific immune activation by peptide stimulation itself over DMSO.

To account for this unspecific immune activation and to be able to compare between groups, we used the stimulation index (SI), calculated as previously reported (11–13) as individual MP response divided by DMSO response. SI in the seropositive group was increased over unexposed controls for R_MP as well as Spike-OS_MP stimulation. Additionally, on Spike-OS_MP stimulation SI was increased in seropositives over seronegative siblings (**Figures 1D, E**).

By applying a SI threshold of > 3 to define responders (11–13), 61% (31 of 51) of seropositive children showed a specific CD4+ T cell response to either Spike-OS_MP or R_MP stimulation, with 55% (28 of 51) being responsive to Spike-OS_MP and 33% (17 of 51) to R_MP. This provides further evidence for the generation of antigen-specific T cells after SARS-CoV-2 infection in children and additionally underscores the dominant immune response elicited by the SARS-CoV-2 Spike protein.

Additionally, reactive T cells were detected in 8% (2 of 24 Spike-OS_MP) and 13% (3 of 23 R-MP) of seronegative siblings, as well as in 14% (7 of 51 Spike-OS_MP) and 12% (6 of 51 R_MP) of unexposed controls.

Without detectable differences in T cell response between seronegative siblings and unexposed controls, our data indicate that the intensity of exposure to SARS-CoV-2 in household members, which did not lead to a humoral immune response (seronegative siblings) was generally also not sufficient to induce a systemic CD4+ T cell response.

Naive versus memory phenotype of AIM+ CD4+ T cells was determined using CD27 and CD45RA expression (**Figure S1A**). This revealed that SARS-CoV-2 specific AIM+ CD4+ T cells predominantly exhibited a central memory phenotype. The proportion of naive cells within the AIM+ fraction was, however, higher in younger children paralleling the higher naive fraction in total CD4+ cells (Spike – OS_MP in **Figures 1F, G**, R_MP and Spike – BV_MP in **Figure S2**).

### Cross-Reactivity to SARS-CoV-2 Beta Variant After Infection With an Ancestral Variant

Occurrence of new SARS-CoV-2 variants of concern, characterized by higher transmissibility and a certain immune escape has changed the pandemic’s dynamic several times, reviewed in (21). It is therefore of great interest whether T cell responses to earlier SARS-CoV-2 infections or the current vaccinations approved in children, which are based on the original Wuhan-Hu-1 strain by either containing inactivated virus (22, 23) or the Spike protein sequence (24, 25), lead to generation of cross-reactive CD4+ T cells against new SARS-CoV-2 variants.

**Figure 2:**
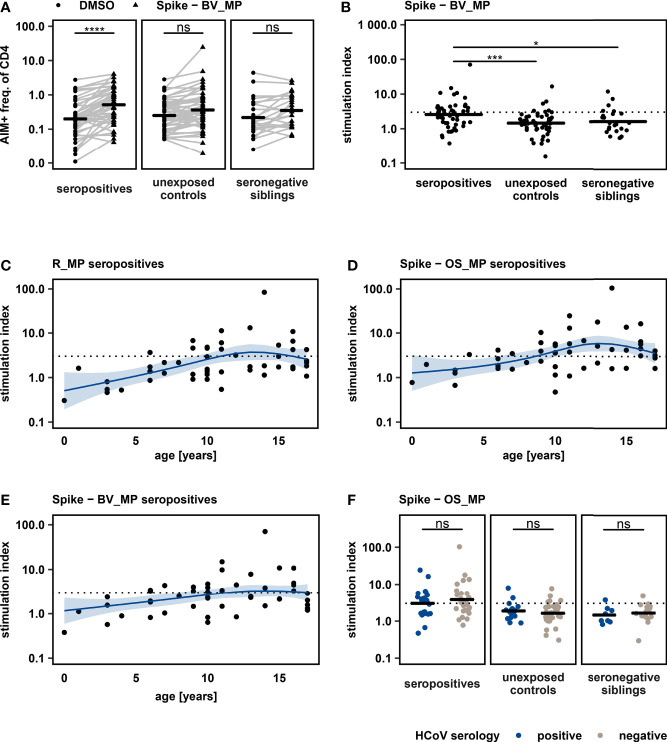
**(A)**: Frequency of AIM+ cells after stimulating PBMC with Spike – BV_MP and DMSO negative control, with horizontal lines showing mean values. One sided paired t – tests were used to quantify P – values. **(B)**: Comparison of T cell response, quantified by stimulation index, to Spike – BV_MP peptide pool between study groups. Mean values are represented by horizontal lines. Statistical analysis was performed with one way ANOVA followed by pairwise t – tests. SI > 3 (dashed line) was defined as response. **(C + D + E)**: T cell responses upon peptide stimulation as quantified by stimulation index are displayed according to the participants’ age. Each dot represents the T cell response of an individual seropositive study participant. The respective peptide pool is indicated on top of each graph. The effect of age on the magnitude of T cell response, was further analyzed with a non-parametric multivariate regression analysis, by using a spline model (blue lines with light blue areas indicating 95% confidence intervals). **(F)**: T cell responses towards Spike – OS_MP stimulation were compared between participants with different serostatus for “common cold” coronaviruses (HCoV, strains 229E, NL63, OC43 and HKU1). Analyses were conducted within study groups, which were defined by SARS-CoV-2 exposure and serostatus. Horizontal lines represent mean values. Unpaired t – test was used to quantify P values. *P < 0.05, ***P < 0.001, ****P < 0.0001, ns, not significant.

We evaluated the T cell response towards the beta variant, a WHO variant of concern due to its capability to escape humoral immunity (26), in archived pediatric samples after infection with an ancestral SARS-CoV-2 variant. PBMC were stimulated with a peptide MegaPool of overlapping peptides spanning the entire Spike glycoprotein of the beta variant (Spike-BV_MP). This resulted in a significant increase of CD4+ AIM markers in the seropositive cohort (**Figure 2A**). 45% of seropositives (23 of 51), 12% of unexposed controls (6 of 51) and 21% of seronegative siblings (5 of 24) could be identified as responders (SI >3). SI was significantly higher in seropositive individuals when compared to unexposed controls and seronegative siblings (**Figure 2B**). Our results suggest that prior infection with an ancestral SARS-CoV-2 variant is associated with the generation of T cells showing reactivity against the immune escaping SARS-CoV-2 beta variant in a relevant proportion of children.

### Lower Antigen-Specific T Cell Detection Rate in Very Young Children

We analyzed the effect of age on SARS-CoV-2 specific CD4+ T cell responses (**Figures 2C–E**), by applying a non-parametric spline model to fit the data on T cell responses as measured by SI plotted by age in years. This model predicts an increase in antigen specific CD4+ T cell response with age, until a plateau is reached between ten and fifteen years. Also, we detected an impaired capacity to mount specific T cell responses (SI > 3) in preschool children using all tested SARS-CoV-2-derived peptide pools. At the same time, we did not detect an overall reduced capacity of CD4+ T cells in younger children to express AIMs upon activation in the positive control (PHA, **Figure S4**).

### Influence of Prior HCoV Infections

To evaluate the possible influence of T cell cross reactivity elicited by prior infections with a common cold Coronavirus (HCoV), we assessed the serostatus of HCoV strains 229E, NL63, OC43 and HKU1 in all study participants. 40% of the participants in the study were identified as seropositive for at least one of the tested strains. When comparing T cell response quantified as SI for stimulation with R_MP, Spike – OS_MP and Spike – BV_MP between participants with positive and negative HCoV serology, no difference could be seen regardless of SARS-CoV-2 serostatus (Spike – OS_MP **Figure 2F** blue and grey, R_MP and Spike – BV_MP in **Figure S3**). Some, but not all unexposed controls and seronegative siblings with SI > 3 in any of the peptide pools had a positive HCoV serology. This indicates that the identified SARS-CoV-2 reactive T cells in seronegative siblings and unexposed controls cannot be explained only by cross reactive T cell memory from prior HCoV infections.

### Cytokine Profile of SARS-CoV-2-Reactive T Cells

To further characterize the T cell response, thirteen different cytokines were measured in culture supernatants after exposure of PBMC to SARS-CoV-2 peptide MegaPools. To be able to attribute cytokine secretion to a specific T cell response and to control for cytokine secretion elicited by unspecific or bystander immune activation, cytokine responses were compared between seropositive children with a clear T cell response (SI > 3 as quantified using activation induced markers) and unexposed controls and seropositive children both without a clear T cell response (SI < 3). Cytokine responses were quantified as fold increase after peptide stimulation over DMSO negative control to correct for variance of the number of available stimulated PBMC between samples.

**Figure 3:**
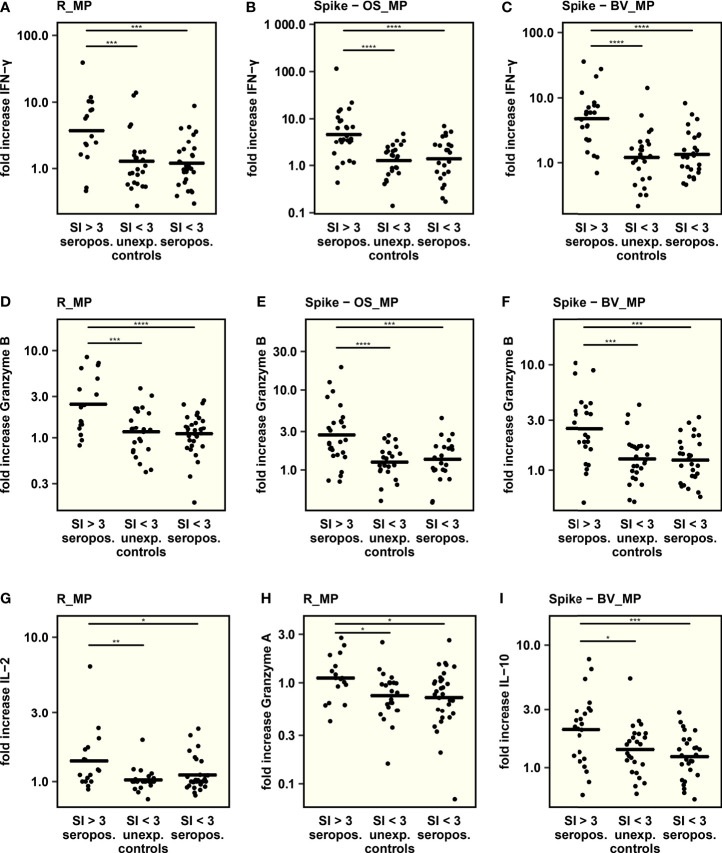
The concentration of 13 cytokines was determined in cell culture supernatants after stimulation with SARS-CoV-2-derived peptide MegaPools. The respective peptide pool, used for stimulation, is indicated on top of each graph. Comparison was conducted between seropositive children with and without a clear T cell response and unexposed controls without a T cell response. T cell response was defined as a stimulation index (SI) > 3 in the AIM assay upon peptide stimulation. **(A–I)**: Comparison of IFN-γ, Granzyme B, IL-2, Granzyme A and IL-10 secretion between groups. To adjust for varying numbers of stimulated PBMCs and unspecific cytokine secretion, comparison was performed by using the fold increase in cytokine concentration after peptide stimulation over cytokine concentration in DMSO treated samples. Mean values are indicated by horizontal lines. One way ANOVA and *post hoc* pairwise t – tests were used to quantify P values. *P < 0.05, **P < 0.01, ***P < 0.001, ****P < 0.0001, not significant – not displayed.

The analysis revealed that levels of IFN-γ and Granzyme B, both associated with viral defense, were consistently higher in seropositive T cell responders after Spike-OS_MP, R_MP and Spike-BV_MP stimulation compared to seropositives and unexposed controls both without a T cell response (**Figures 3A–F**). Since PBMC were stimulated in bulk, the elevated IFN-γ and especially Granzyme B levels could indicate concomitant CD8+ activation.

In seropositive T cell responders, R_MP stimulation led to an increased secretion of IL-2 and Granzyme A (**Figures 3G, H**, **S7**). Interestingly, the beta-variant-based Spike-BV_MP was the only peptide pool eliciting the secretion of anti-inflammatory IL-10 in association with a CD4+ T cell response (**Figures 3I**, **S7**). The rest of the tested cytokines IL-4, IL-6, IL-17A, TNF-α, sFas, sFasL, Perforin and Granulysin did not show specific differences associated with the CD4+ T cell response.

### No Long-Term Alterations of the Immune Phenotype in Convalescent Pediatric Patients

**Figure 4:**
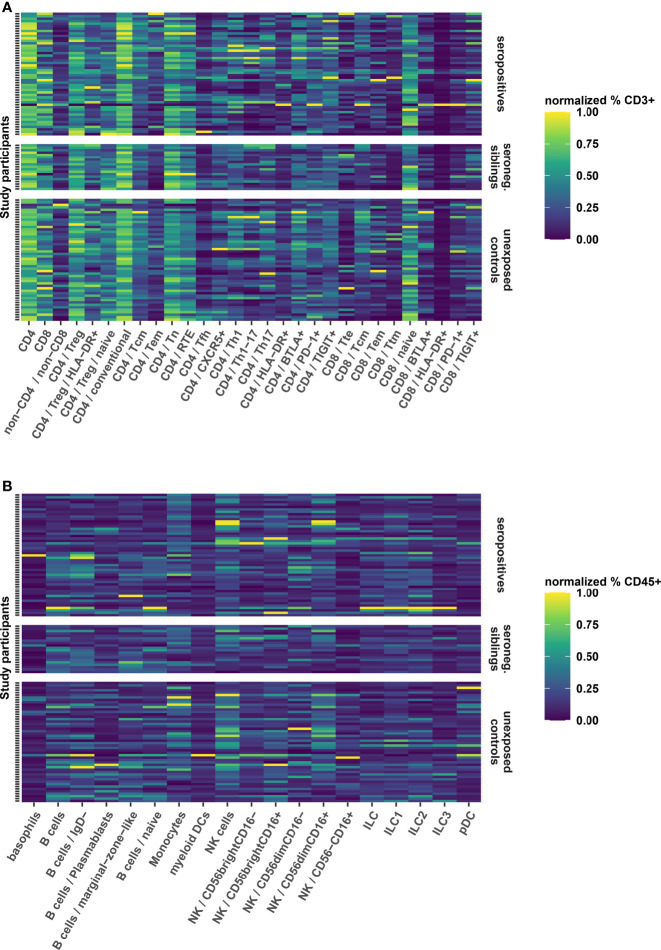
Heatmaps showing the relative frequency of T cell subsets **(A)** as frequency of CD3 positive live cells and B- and innate cell subsets **(B)** as frequency of CD45 positive live cells. Further subdivision of subsets is indicated by forward slash “/”, whereby the parent population is indicated in front of the child population. The frequencies have been normalized for each cellular subset to utilize the full color scale, which is indicated to the right of both panels. Each row represents a PBMC sample from a single participant. Samples are grouped according to seroconversion status (right y axis). Furthermore, samples within groups are sorted vertically according to the age of the participant, beginning with the oldest (top) to the youngest (bottom). MAIT, Mucosa Associated Invariant T cells; Treg, regulatory T cells; CD4 conventional, all non-Treg CD4+ cells; Tcm, central memory T cells; Tem, effector memory T cells; Tn, naive T cells; RTE, recent thymic emigrants; Tfh, T follicular helper cells; Th, T helper cells; HLA, human leukocyte antigen; BTLA, B- and T-lymphocyte attenuator; PD-1, programmed death 1; TIGIT, T cell immunoreceptor with Ig and ITIM domains; Tte, terminally differentiated effector T cell; Ttm, transitional memory T cell; pDC, plasmacytoid dendritic cells; NK, natural killer cells; ILC, innate lymphoid cells.

Respiratory infections are known to elicit long-term changes in the phenotype of innate and adaptive immune cell populations (27, 28). Therefore, we performed a broad immunologic phenotyping of study participants in parallel PBMC samples using two flow cytometry panels for a detailed T cell profiling and quantification of main immune cell subsets, respectively (**Figures S1B, C**). We found that the relative abundance of T-, B- and innate cell subsets was similar in seropositive children, seronegative siblings and unexposed controls (**Figures 4A, B**). Thus, in this pediatric cohort, no long-term immune phenotypic changes after SARS-CoV-2 infection or exposure could be demonstrated.

## Discussion

Antigen specific CD4+ T cells play a central role in the immune response to a viral infection, as they may reduce disease severity after re-infection by a more rapid clearance of the virus and better overall disease control, as reviewed in (29). The presence of antigen specific T cells after SARS-CoV-2 infection in adult cohorts has been widely demonstrated (8–10, 12, 13, 30–32). Here, we analyzed the T cell response in a large cohort of SARS-CoV-2 convalescent children, their seronegative siblings as well as unexposed controls. We provided further evidence that a high proportion of children who seroconverted to SARS-CoV-2 were able to mount specific CD4+ T cell responses, that were still detectable up to over 100 days after infection. In contrast to adult cohorts (33) we could identify specific T cell responses in only a minority of SARS-CoV-2 exposed but not seroconverted children (seronegative siblings). In our pediatric cohort we showed a T cell response attributable to a prior SARS-CoV-2 infection in 61% of seropositive children, which is less than the reported response rates of 77 – 100% of acutely ill (8, 9, 12) and 81 – 100% of convalescent adult patients (7, 9, 13, 31, 32). These findings are in line with previous studies investigating systemic T cell responses in children after respiratory infections like SARS-CoV-2 (17, 18) or Influenza (34).

Until now, data on pediatric T cell response after SARS-CoV-2 infection is scarce with cohort sizes not allowing for analysis over different age groups (17, 18). Importantly, we showed an increase in SARS-CoV-2-specific CD4+ T cell responses by age within this pediatric cohort. Reduced capacity to mount specific T cell responses was particularly seen in preschool children. A comparable age-effect showing lower T cell responses after SARS-CoV-2-infection in children than adults has previously been demonstrated (18). Such age related differences have been reported, especially in infants, regarding the interplay between innate and adaptive immunity and the development of memory T cell responses including a lower inflammatory reaction upon pathogen challenge and vaccination (35, 36).

We provided functional data on the phenotype of the pediatric CD4+ T cell response to SARS-CoV-2 in children showing a strong antiviral response and provide indirect evidence for a cytotoxic CD8+ response involving Granzyme B production. Predominant CD4+ T cell response in convalescent children is likely of Th1 bias (IFN-γ).

While a large proportion of seroconverted children appeared to mount strong T cell responses, long-term changes in the immunological phenotype of innate and adaptive cell populations seem not to affect children after mild SARS-CoV-2 infection. Notably, children in this cohort were typically sampled about three months after COVID-19, with only about half of those seropositive and exposed having exhibited symptoms of infection in the first place. These characteristics may explain the quick normalization of any immunological changes post infection in this cohort. Conversely, in cohorts with a larger proportion of symptomatic or seriously ill children, also CD4+ T cell reactivity could be higher.

Cross-reactive T cells have the potential to reduce disease severity of infections with new viral strains, reviewed in (37, 38). The presence of SARS-CoV-2 reactive CD4+ T cells in unexposed individuals could be shown (7, 8, 12, 31, 32) and cross reactivity of CD4+ T cells to epitopes of HCoVs and SARS-CoV-2 was demonstrated (39). We analyzed the influence of prior HCoV infections on pediatric SARS-CoV-2 T cell response. In our cohort, we could not detect a difference in the CD4+ T cell response (quantified by SI) upon SARS-CoV-2-derived peptide stimulation between HCoV seropositive and seronegative individuals. Also, SARS-CoV-2-reactive T cells in unexposed controls were only present in a fraction of cases associated with a positive HCoV serology. We observed a seropositivity for HCoV in 49% of SARS-CoV-2 seropositive and 33% of SARS-CoV-2 exposed participants while no severe disease courses were reported in our cohort. It would follow, that the comparatively mild course of COVID-19 in children is not mainly or exclusively explained by their more frequent or recent exposures to related human coronaviruses. More likely, reduced disease severity and lower T cell responses in children are offset by an enhanced capability of innate, mucosal and tissue resident immune responses in the upper airways resulting in early SARS-CoV-2 control in this age group (40).

Importantly, by making use of our relatively large cohort of archived pediatric samples, we demonstrated for the first time to our knowledge a cross reactivity of T cells after infection with an ancestral SARS-CoV-2 variant to an immune escaping SARS-CoV-2 variant of concern (B.1.351-beta variant) in a pediatric population. This adds on to previous findings, describing cross reactive T cells to B.1.351, B.1.1.7 (alpha-variant), P.1 (gamma-variant) B.1.617.2 (delta-variant), B.1427/9 (epsilon-variant) and B.1.1.529 (omicron-variant) in adult cohorts after SARS-CoV-2 infection or vaccination (15, 16, 32, 41–43). T cell response to the beta variant may serve as a model for immune escaping variants, as immune escape is mainly attributed to its mutation on position E484 within the spike domain, which is associated with reduced affinity of neutralizing antibodies (44) and mutations on this site are also found in SARS-CoV-2 strains B.1.617.2 (gamma), B.1.621 (mu) or B.1.1.529 (omicron) (45).

Our data provide important evidence for a certain cross reactivity of the pediatric CD4+ T cell response after infection with an ancestral SARS-CoV-2 variant to mutations in the Spike domain. Current SARS-CoV-2 vaccines approved for use in children, consisting of inactivated virus (22, 23) or the genetic information of the Spike protein (24, 25), are based on the original SARS-CoV-2 Wuhan-Hu-1 strain. Based on our data generated by stimulation of archived samples from the first wave with peptide MegaPools derived from the later evolved beta variant, we can hypothesize that these vaccines should still elicit adequate T cell responses to emerging immune escaping variants in children. CD4+ T cell responses in preschool children were reduced, which may be offset by antibody responses (46) or increased innate responses (40). Nonetheless, the role of a lower CD4+ memory T cell response in the youngest should be considered when planning vaccination strategies and if emerging viral variants cause a rise in pediatric disease burden.

Our study had several limitations. As children were recruited weeks or months after infection, no PCR based confirmation from the acute phase of infection was available. As previous infection was defined through seropositivity alone, a combined positivity in three independent serological tests was required and this approach should minimize the possibility of false positives. A further limitation based on the retrospective study design is that a detailed history from the time of infection as well as the exact time of infection could only be collected for a fraction of participants. Because of a reluctance in small children and their caregivers, as well as the technical difficulty regarding blood draws, younger age groups were relatively underrepresented. Also, CD8+ T cell responses were not investigated - these could provide important insights into cellular anti-SARS-CoV-2 immunity and should be the focus of further studies.

Here we showed that pediatric CD4+ T cell responses after infection with an ancestral SARS-CoV-2 variant are age dependent, with reduced capability of the youngest to mount specific responses. Antigen specific T cells persist over three months after infection and are cross reactive with the SARS-CoV-2 variant of concern B.1.351-beta variant. We detected a strong antiviral cytokine response in association with SARS-CoV-2-specific T cell activation. Our findings have relevance when planning rational vaccination of children as well as social distancing measures involving the pediatric population in case of emerging SARS-CoV-2 variants.

## Data Availability Statement

The raw data supporting the conclusions of this article will be made available by the authors, without undue reservation.

## Ethics Statement

The studies involving human participants were reviewed and approved by Ethik-Kommission, Ärztekammer Hamburg, Weidestr. 122 b, 22083 Hamburg, Germany. Written informed consent to participate in this study was provided by the participants’ legal guardian/next of kin.

## Author Contributions

KP, SG and GD conceived the study. KP, FS and GD performed T cell stimulation assays, cytokine analysis and immune phenotyping. ML supervised serology testing. KP, FS, DW, MB, AS, ET, JS, ML, SG and GD contributed to the experiment design and methodology. JO, TM, AM, SG, GD were responsible for study supervision and funding. KP, MW, KH, AK, DZ, LH, MD, JO, TM, AM, GD coordinated the study cohort and acquired patient samples. Sample preparation was supervised by KP, FS and GD. KP, GD, LG, ET, EV, and AZ performed data- and statistical analysis and prepared graphs. All authors provided conceptual input. KP, FS and GD wrote the manuscript, which was critically revised by all authors.

## Funding

The C19.CHILD Hamburg Study received funding from the Senate Chancellery of the Free and Hanseatic City of Hamburg. The following foundations and organizations have provided financial support: Carlsen Verlag, Dr. Melitta Berkemann Stiftung, Fördergemeinschaft Kinderkrebs‐Zentrum Hamburg e.V., Freunde der Kinderklinik des UK Eppendorf e.V., HSV Fussball AG, Joachim‐Herz‐Stiftung, Michael Otto Stiftung, Michael Stich Stiftung, Nutricia, Stiftung KinderHerz, EAGLES Charity Golf Club e.V., DAMP Stiftung, Kroschke Stiftung, ZEIT‐Stiftung. The funders were not involved in the study design, collection, analysis, interpretation of data, the writing of this article or the decision to submit it for publication.

## Conflict of Interest

The authors declare that the research was conducted in the absence of any commercial or financial relationships that could be construed as a potential conflict of interest.

## Publisher’s Note

All claims expressed in this article are solely those of the authors and do not necessarily represent those of their affiliated organizations, or those of the publisher, the editors and the reviewers. Any product that may be evaluated in this article, or claim that may be made by its manufacturer, is not guaranteed or endorsed by the publisher.
