# Supplementary material for: Specific CD4+ T Cell Responses to Ancestral SARS-CoV-2 in Children Increase With Age and Show Cross-Reactivity to Beta Variant
Source: Front Immunol. 2022 Jul 15;13:867577. doi: 10.3389/fimmu.2022.867577 (PMC9336222; doi:10.3389/fimmu.2022.867577)
Supplement: Supplementary file 1 [file DataSheet_1.docx]

Specific CD4+ T cell responses to ancestral SARS-CoV-2 in children increase with age and show cross-reactivity to beta variant

Supplementary Material

Contents

[Supplementary Table 1. Flow cytometry reagent list. 2](#_Toc99196319)

[Supplementary Table 2. HCoV serology results by strain and study group. 4](#_Toc99196320)

[Supplementary Figure 1A. Gating strategy for activation induced markers “Panel AIM” 5](#_Toc99196321)

[Supplementary Figure 1B. Gating strategy for immune phenotyping, “T cells” 6](#_Toc99196322)

[Supplementary Figure 1C. Gating strategy for immune phenotyping, “B cells and innate immune cells” 7](#_Toc99196323)

[Supplementary Figure 2. Memory phenotypes upon peptide stimulation 8](#_Toc99196324)

[Supplementary Figure 3. T cell response to peptide stimulation compared according to HCoV serostatus within study groups 9](#_Toc99196325)

[Supplementary Figure 4. T cell response to stimulation by PHA-L (positive control) according to experimental groups and age 10](#_Toc99196326)

[Supplementary Figure 5. Antibody reactivity towards HCoV strains by study group 11](#_Toc99196327)

[Supplementary Figure 6. Comparison of T cell response to peptide stimulation, quantified as AIM+ freq. of CD4 after DMSO background subtraction 12](#_Toc99196328)

[Supplementary Figure 7. Comparison of secreted IL-2, Granzyme A and IL-10 towards peptide stimulation between groups. 13](#_Toc99196329)

# Supplementary Table 1. Flow cytometry reagent list.

| Antigen | TAG | Clone | Supplier | #Cat | Purpose |
| --- | --- | --- | --- | --- | --- |
| BTLA | BV421 | MIH26 | Biolegend | 344512 | Coinhibitory receptor |
| CCR6 | APC | G034E3 | Biolegend | 353416 | Th17 cells |
| CD117 | APC | 104D2 | Biolegend | 313206 | c-kit, ILC3 |
| CD123 | PE-Cy7 | 6H6 | Biolegend | 306010 | Interleukin-3 receptor, pDC |
| CD127 | BUV 737 | HIL-7R-M21 | BD | 612794 | Interleukin-7 Receptor, Treg |
| CD134 (OX40) | PE-Cy7 | Ber-ACT35 | Biolegend | 350012 | activation |
| CD137 | APC | 4B4-1 | Biolegend | 309809 | activation |
| CD14 | BUV 563 | M5E2 | BD | 741360 | Lineage, monocytes |
| CD16 | BV605 | 3G8 | Biolegend | 302039 | Fc-Receptor, monocytes / NK cells |
| CD161 | BV421 | HP-3G10 | Biolegend | 339914 | C-type lectin, ILC1 |
| CD19 | AF700 | SJ25C1 | Biolegend | 363034 | Lineage, B cells |
| CD25 | PE | M-A251 | Biolegend | 356104 | IL-2 receptor, activation, Treg |
| CD27 | BV650 | O323 | Biolegend | 302827 | TNF-receptor, differentiation |
| CD27 | BV421 | O323 | Biolegend | 302824 | Differentiation |
| CD28 | AF700 | CD28.2 | Biolegend | 302920 | Costimulatory receptor, effector cells |
| CD294 (CRTH2) | PE | BM16 | Biolegend | 350106 | Prostaglandin D2 receptor, ILC2 |
| CD3 | BV785 | OKT3_ | Biolegend | 317330 | Lineage, T cells |
| CD31 | BV605 | WM59 | Biolegend | 303122 | PECAM-1, CD4+ recent thymic emigrants |
| CD38 | PerCp-Cy5.5 | HIT2 | Biolegend | 303521 | Activation, plasma cells |
| CD4 | BUV 395 | SK-3 | BD | 563552 | Lineage, T cells |
| CD4 | BV605 | SK-3 | Biolegend | 344646 | Lineage, T cells |
| CD45 | BUV 395 | HI30 | BD | 563792 | Common Leukocyte Antigen |
| CD45RA | BV510 | HI100 | Biolegend | 304142 | Differentiation, naive cells |
| CD56 | PE-Dazzle | 5.1H11 | Biolegend | 362544 | Lineage, NK cells |
| CD69 | AF488 | FN50 | Biolegend | 310903 | activation |
| CD8 | BUV 563 | RPA-T8 | BD | 612914 | Lineage, T cells |
| CD8 | AF700 | RPA-T8 | Biolegend | 301028 | Lineage, T cells |
| CXCR3 | PE-Cy7 | G025H7 | Biolegend | 353719 | Th1 cells |
| CXCR5 | PerCp-Cy5.5 | J252D4 | Biolegend | 356910 | Germinal center trafficking, T follicular helper cells |
| HLA-DR | FITC | G46-6 | BD | 555811 | Activation, exhaustion |
| IgD | BV510 | IA6-2 | Biolegend | 348220 | Differentiation, B cells |
| LiveDead DUMP | near-infrared | NA | Life | L10119 | Viability (exclusion) |
| PD-1 | BV711 | EH12.2H7 | Biolegend | 329928 | Co-inhibitory receptor, T follicular helper cells |
| TIGIT | PE-Dazzle | A15153G | Biolegend | 372716 | Coinhibitory receptor |

# Supplementary Table 2. HCoV serology results by strain and study group.

| study Group | 229E | NL63 | OC43 | HKU1 | any HCoV | n total |
| --- | --- | --- | --- | --- | --- | --- |
| Seropositives  positive n (%) | 9 (18%) | 22 (43%) | 4 (8%) | 4 (8%) | 25 (49%) | 51 |
| Seronegative Siblings  positive n (%) | 2 (8%) | 4 (17%) | 3 (13%) | 1 (4%) | 8 (33%) | 24 |
| Unexposed Controls  positive n (%) | 7 (14%) | 11 (22%) | 9 (18%) | 7 (14%) | 18 (35%) | 51 |
| All study Participants  positive n (%) | 18 (14%) | 35 (20%) | 16 (13%) | 12 (10%) | 51 (40%) | 126 |

# Supplementary Figure 1A. Gating strategy for activation induced markers “Panel AIM”


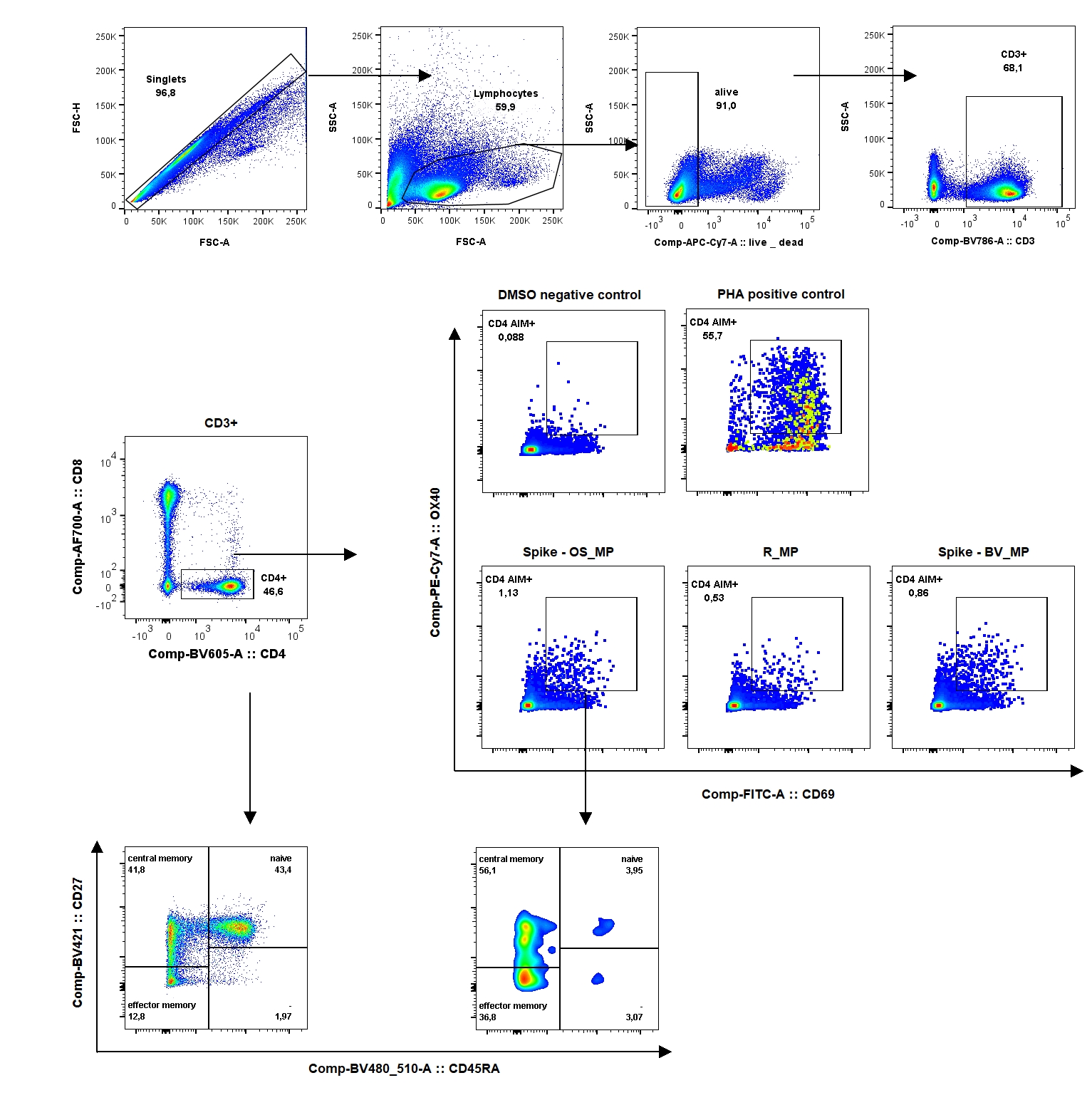
Manual gating strategy for the gating of activation induced markers. Names of subpopulations are shown along with frequency within the parent population. The parent population is indicated above the plots or by arrows showing backgating. For stimulation, the different conditions (negative, positive controls or peptide MegaPools) are indicated above the plots.

# Supplementary Figure 1B. Gating strategy for immune phenotyping, “T cells”

Manual gating strategy for T cell subpopulations on a representative sample. Names of subpopulations are shown along with frequency within the parent population. The parent population is indicated above the plots or by arrows showing backgating.


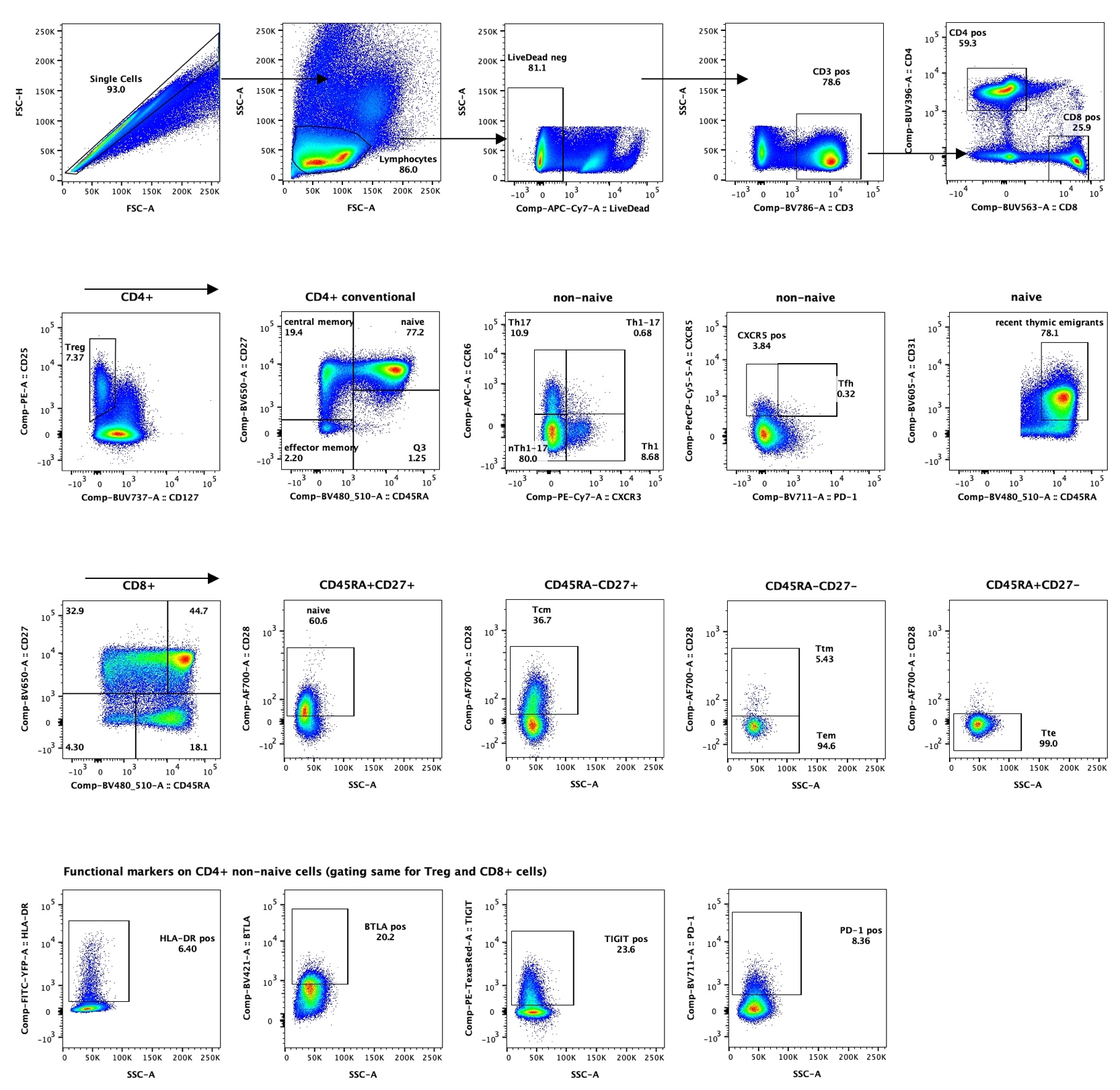


# Supplementary Figure 1C. Gating strategy for immune phenotyping, “B cells and innate immune cells”


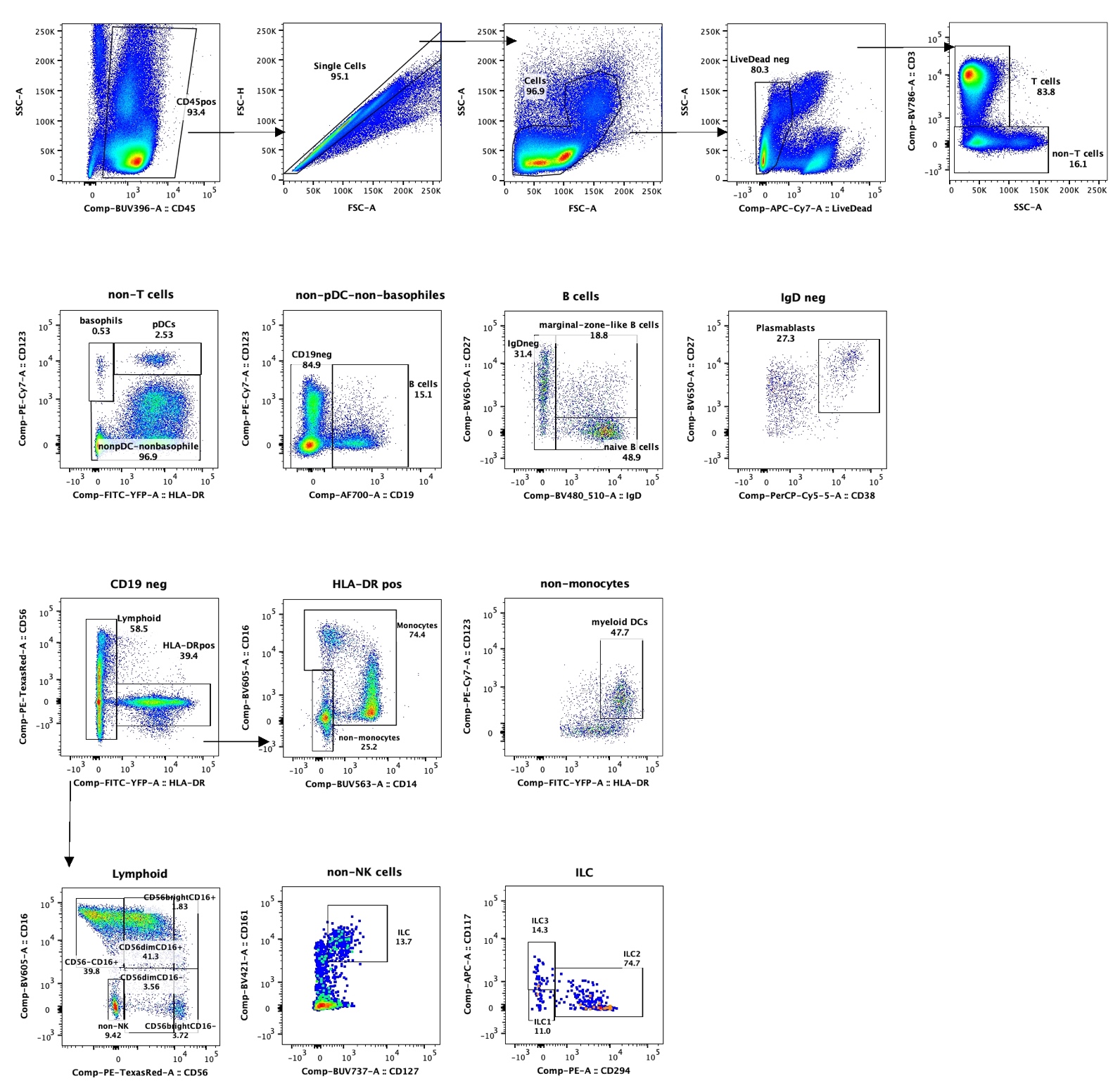
Manual gating strategy for B cells and innate immune cells on a representative sample. Names of subpopulations are shown along with frequency within the parent population. The parent population is indicated above the plots or by arrows showing backgating.

# Supplementary Figure 2. Memory phenotypes upon peptide stimulation

Memory phenotypes of total CD4+ and AIM+CD4+ T cells after peptide stimulation with R_MP **(A + B)** and Spike -BV_MP **(C + D)**. Mean values of all study participants irrespective of SARS-CoV-2 serostatus are displayed.


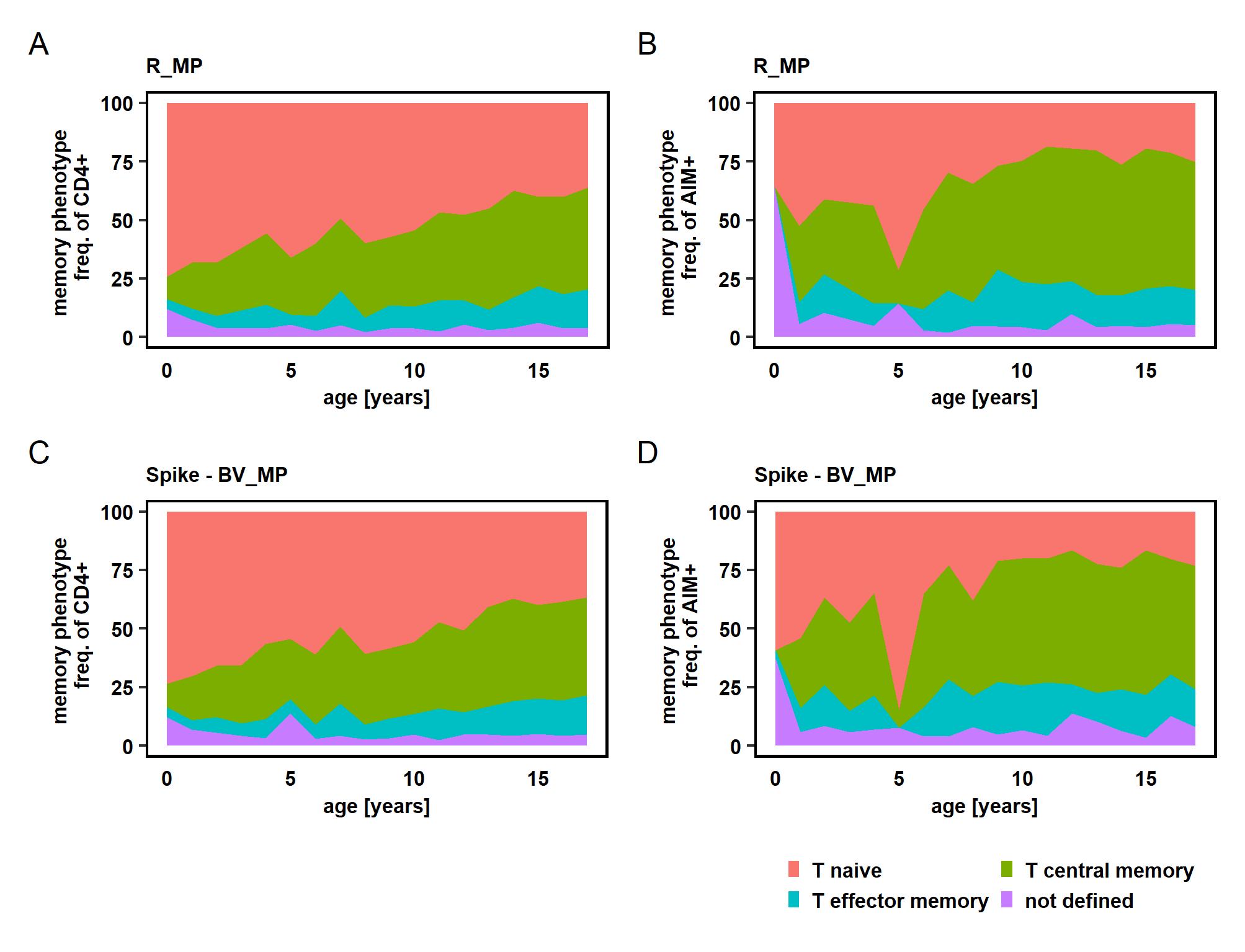


# Supplementary Figure 3. T cell response to peptide stimulation compared according to HCoV serostatus within study groups

Comparison of T cell response towards R_MP **(A)** and Spike – BV_MP **(B)** stimulation between their different serostatus for “common cold” coronaviruses (HCoV). Analyses were conducted within study groups, which were defined by SARS-CoV-2 exposure and serostatus. Unpaired t – test was used to quantify P values. ns - not significant.


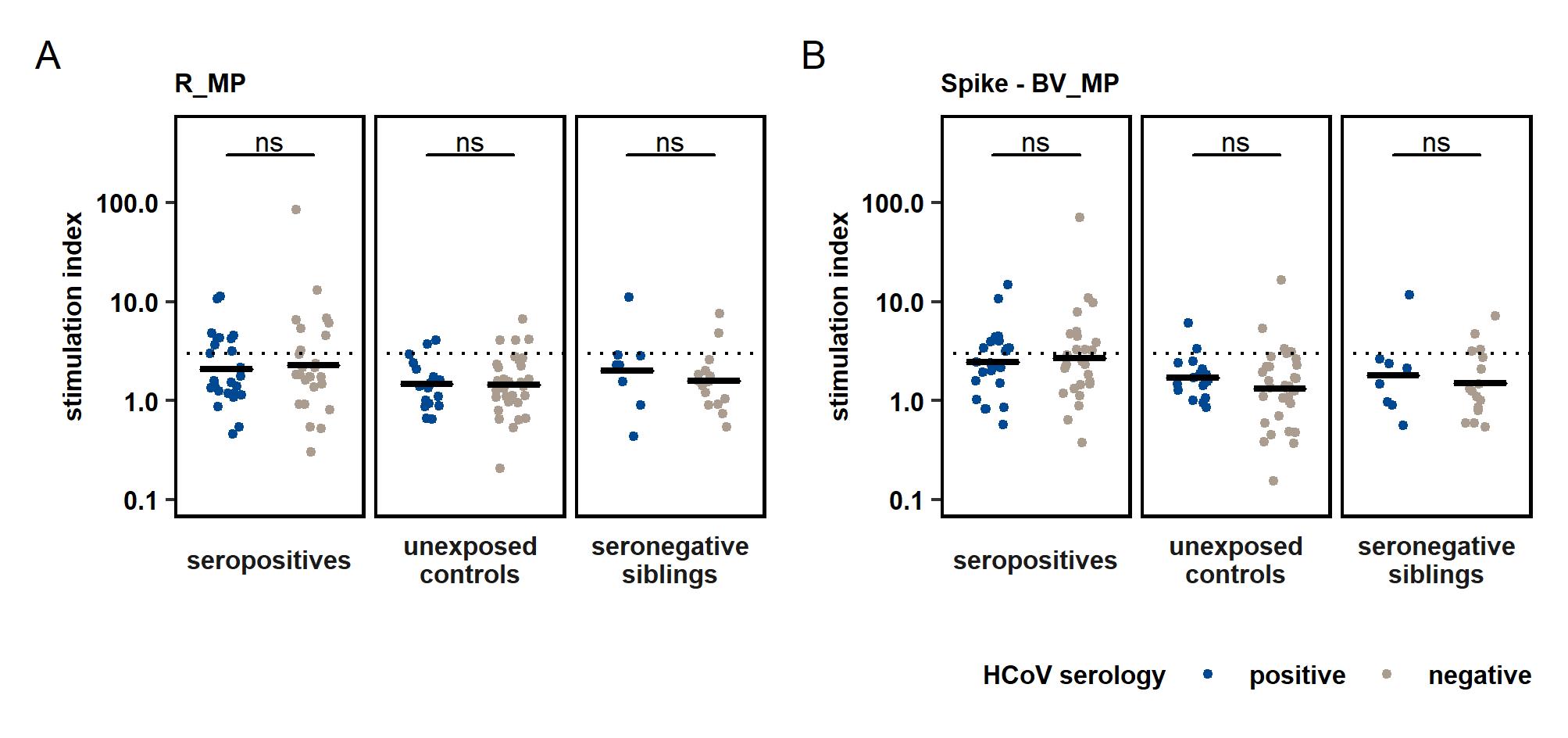


# Supplementary Figure 4. T cell response to stimulation by PHA-L (positive control) according to experimental groups and age

Panels according to experimental groups as indicated on top. T cell responses in the positive control, phytohemagglutinin (PHA-L) are displayed as quantified by stimulation index according to the participants’ age. Dots represent individual T cell responses. The effect of age on the magnitude of T cell response was analyzed with a non-parametric multivariate regression analysis, by using a spline model (blue lines with light blue areas indicating 95% confidence intervals).


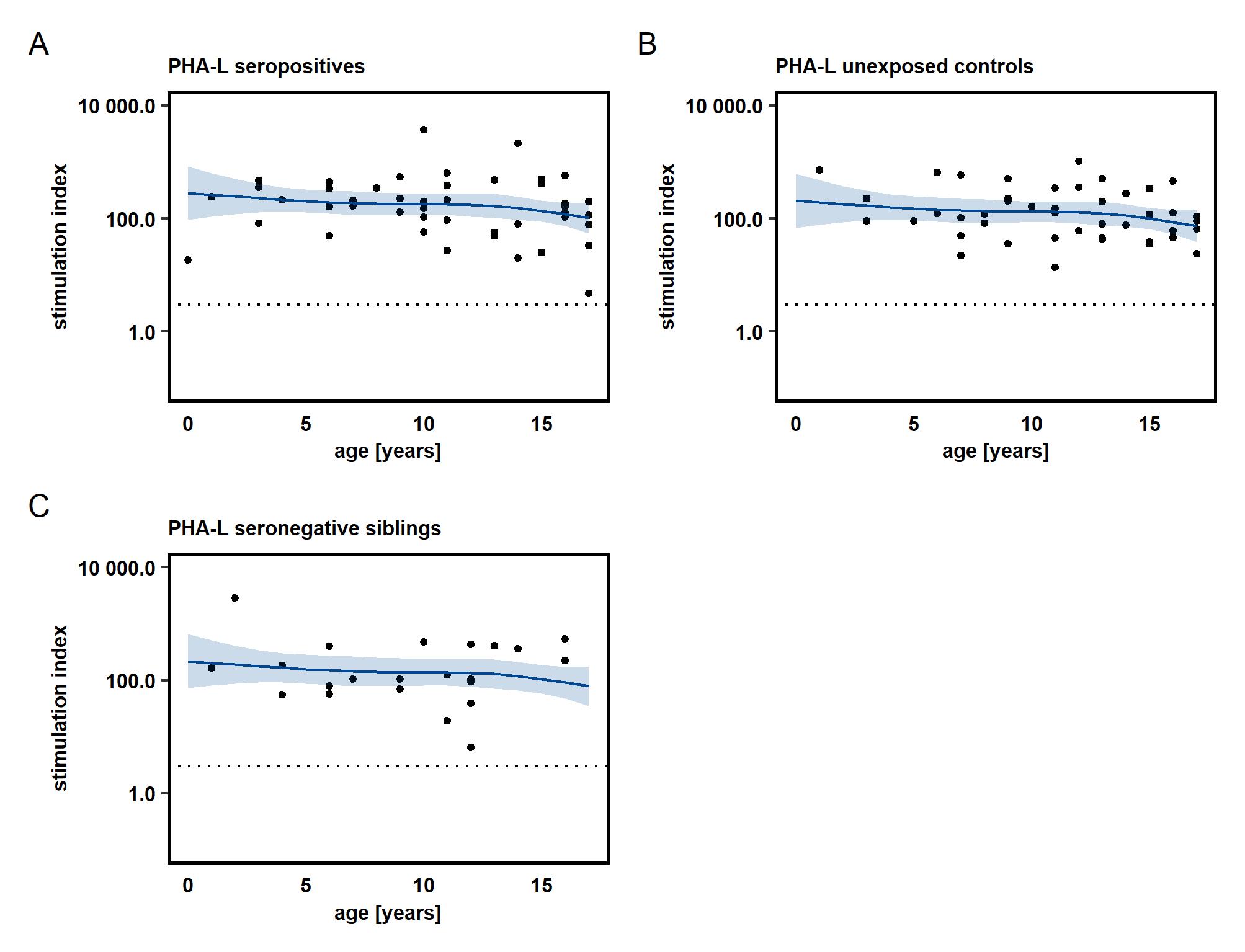


# Supplementary Figure 5. Antibody reactivity towards HCoV strains by study group

Antibody reactivity of the study participants towards different strains of “common cold” corona viruses (HCoV) was evaluated by analyzing serum samples with the recomLine SARS-CoV-2 IgG® assay (Mikrogen) according to manufacturer’s instructions. Level of reactivity was automatically analysed in a semi quantitative manner by comparing the color intensity of the test band to the respective cut off band. A ratio of ≥ 1 (dotted horizontal line) is defined as positive.


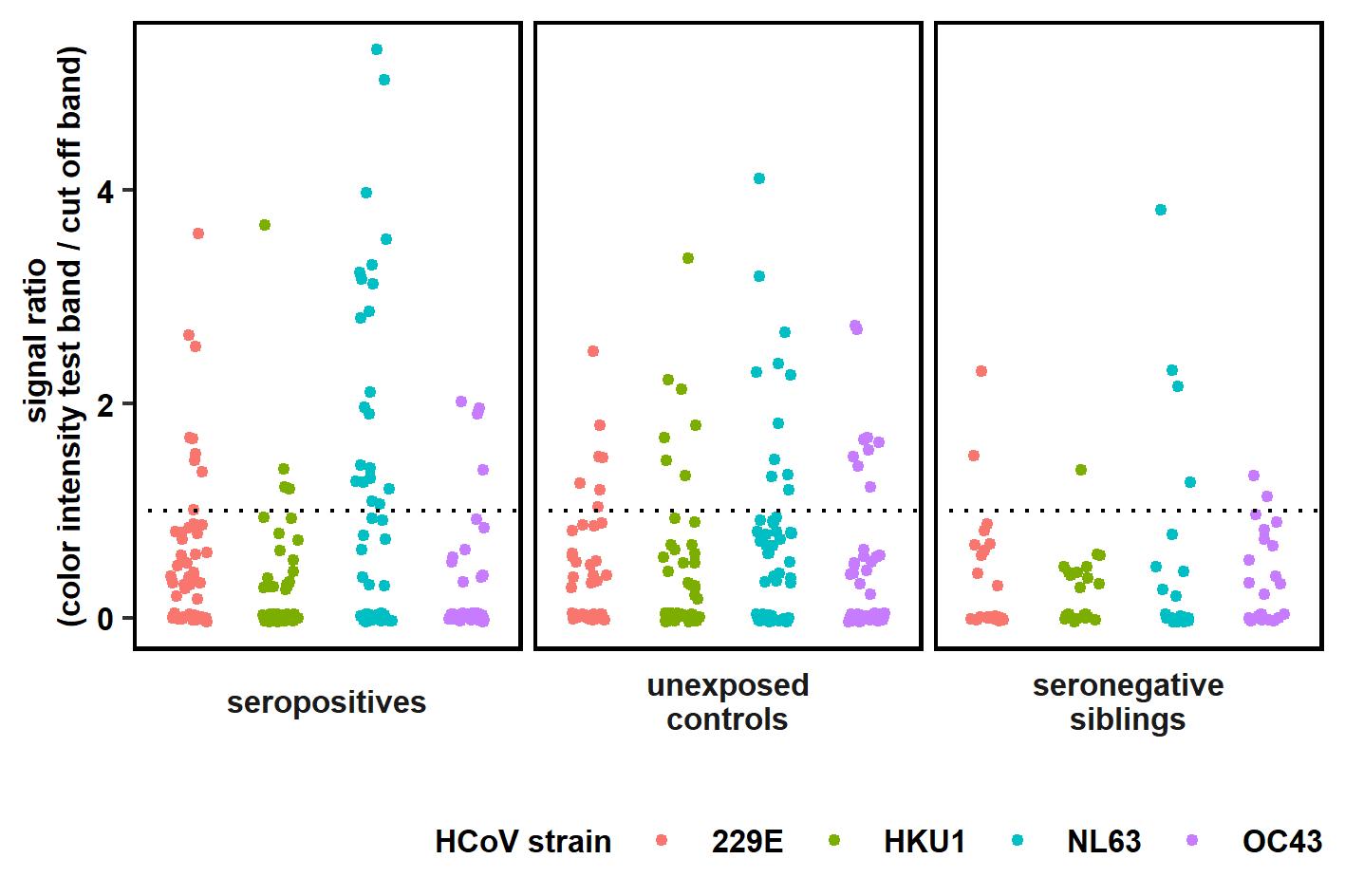


# Supplementary Figure 6. Comparison of T cell response to peptide stimulation, quantified as AIM+ freq. of CD4 after DMSO background subtraction

Panels showing the T cell response towards peptide stimulation as AIM+ (CD69 and OX40 +) freq. of CD4. The used peptide pool or PHA-L positive control are indicated on top of the panels. The background was removed from the data by subtracting AIM+ freq. of CD4 detected in the corresponding DMSO negative control.


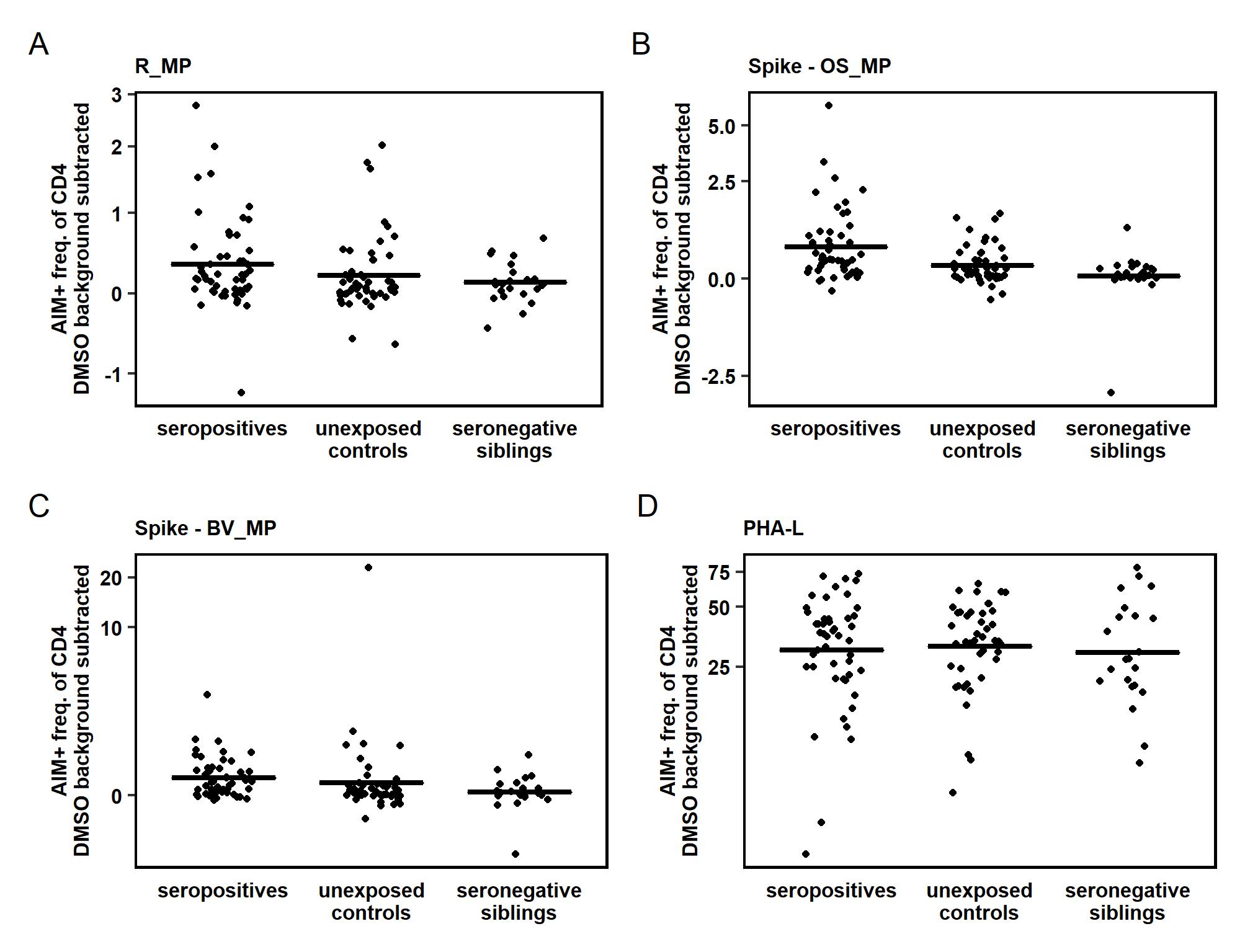


# Supplementary Figure 7. Comparison of secreted IL-2, Granzyme A and IL-10 towards peptide stimulation between groups.

Comparison was performed by using the fold increase in cytokine concentration after peptide stimulation over cytokine concentration in corresponding DMSO treated samples. One way ANOVA and post hoc pairwise t – tests were used to quantify P values.
*P < 0.05, **P < 0.01, ***P < 0.001, ****P < 0.0001, not significant – ns


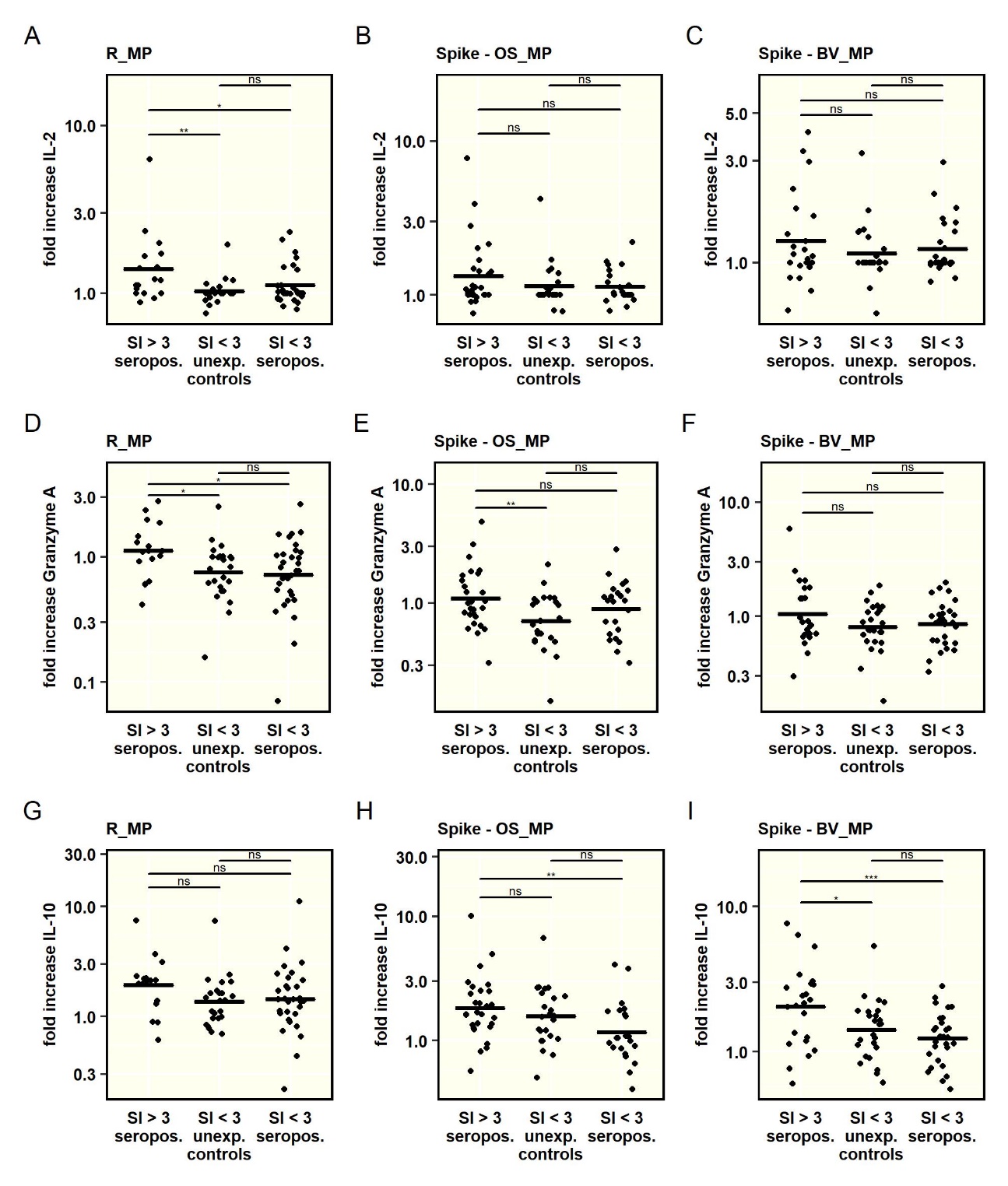


**
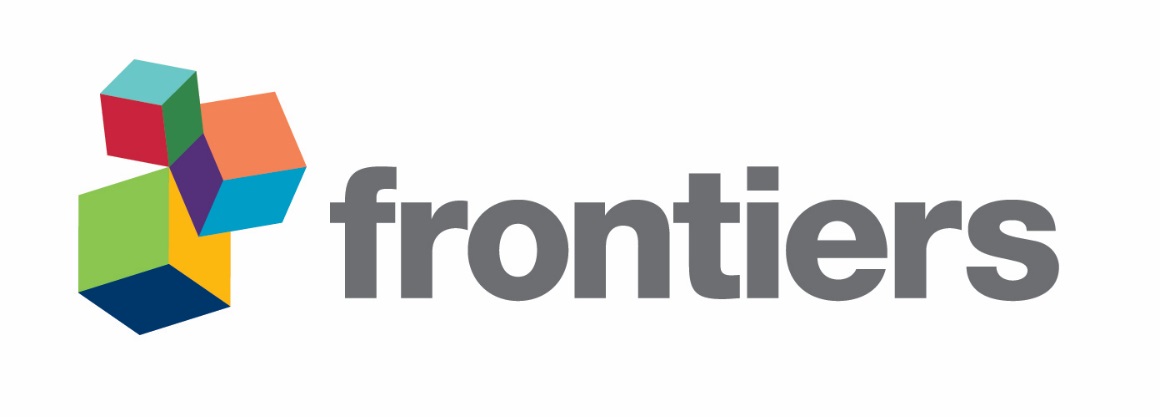
**
